# The *Komagataeibacter europaeus* GqqA is the prototype of a novel bifunctional N-Acyl-homoserine lactone acylase with prephenate dehydratase activity

**DOI:** 10.1038/s41598-021-91536-1

**Published:** 2021-06-10

**Authors:** Nadine Werner, Katrin Petersen, Christel Vollstedt, Pablo Perez Garcia, Jennifer Chow, Manuel Ferrer, Laura Fernandez-Lopez, Sven Falke, Markus Perbandt, Winfried Hinrichs, Christian Betzel, Wolfgang R. Streit

**Affiliations:** 1 Laboratory for Structural Biology of Infection and Inflammation, Institute of Biochemistry and Molecular Biology, University Hamburg, C/O DESY, 22607 Hamburg, Germany; 2 Microbiology and Biotechnology, University Hamburg, 22609 Hamburg, Germany; 3 Institute of Catalysis, Consejo Superior de Investigaciones Científicas, 28049 Madrid, Spain; 4 Institute for Biochemistry, University Greifswald, 17487 Greifswald, Germany

**Keywords:** Microbiology, Structural biology

## Abstract

Previously, we reported the isolation of a quorum quenching protein (QQ), designated GqqA, from *Komagataeibacter europaeus* CECT 8546 that is highly homologous to prephenate dehydratases (PDT) (Valera et al. in Microb Cell Fact 15, 88. 10.1186/s12934-016-0482-y, 2016). GqqA strongly interfered with N-acyl-homoserine lactone (AHL) quorum sensing signals from Gram-negative bacteria and affected biofilm formation in its native host strain *Komagataeibacter europaeus*. Here we present and discuss data identifying GqqA as a novel acylase. ESI–MS–MS data showed unambiguously that GqqA hydrolyzes the amide bond of the acyl side-chain of AHL molecules, but not the lactone ring. Consistent with this observation the protein sequence does not carry a conserved Zn^2+^ binding motif, known to be essential for metal-dependent lactonases, but in fact harboring the typical periplasmatic binding protein domain (PBP domain), acting as catalytic domain. We report structural details for the native structure at 2.5 Å resolution and for a truncated GqqA structure at 1.7 Å. The structures obtained highlight that GqqA acts as a dimer and complementary docking studies indicate that the lactone ring of the substrate binds within a cleft of the PBP domain and interacts with polar residues Y16, S17 and T174. The biochemical and phylogenetic analyses imply that GqqA represents the first member of a novel type of QQ family enzymes.

## Introduction

Quorum sensing (QS) is very well-described process in both Gram-negative and Gram-positive bacteria^2–6^. The term QS describes the cell–cell communication within a bacterial community (mono-and multi-species) in dependence of their cell density. Thereby, bacteria synthesize and release small often diffusible molecules that allow measuring the density of a population. The different signaling molecules are sensed by other bacteria within the species or across the species. The sensing induces the coordinated transcription of genes and proteins that are of importance for the survival of the community but that are not necessarily essential at a single cell level. In general, it is assumed that through the expression of the AI synthase gene at very low level, the signal is constantly produced and not or only poorly sensed until a threshold concentration is reached. Only at higher levels, which goes in parallel with a higher cell density, then the AI can interact with the respective receptors and regulators and induce coordinated gene expression at a population wide level. Genes regulated by this phenomenon are involved in surface colonization and attachment, biofilm formation, virulence factor production, secretion, infection, conjugation, secondary metabolite production, bioluminescence, sporulation and symbiosis^2–6^.

During evolution bacteria developed a variety of different mechanisms using various QS signal molecules. Among these are *N*-acyl-homoserine lactones (AHLs) probably the best studied AI molecules with respect to their influence on a population wide level. AHLs in Gram-negative bacteria consist of an acyl moiety linked to an (*S*)-α-amino-γ-butyrolactone ring via an amide bond. The acyl chain is variable in length and frequently modified by a 3-oxo- or a 3-hydroxy-group to increase the specificity^7–9^.

**Figure 1:**
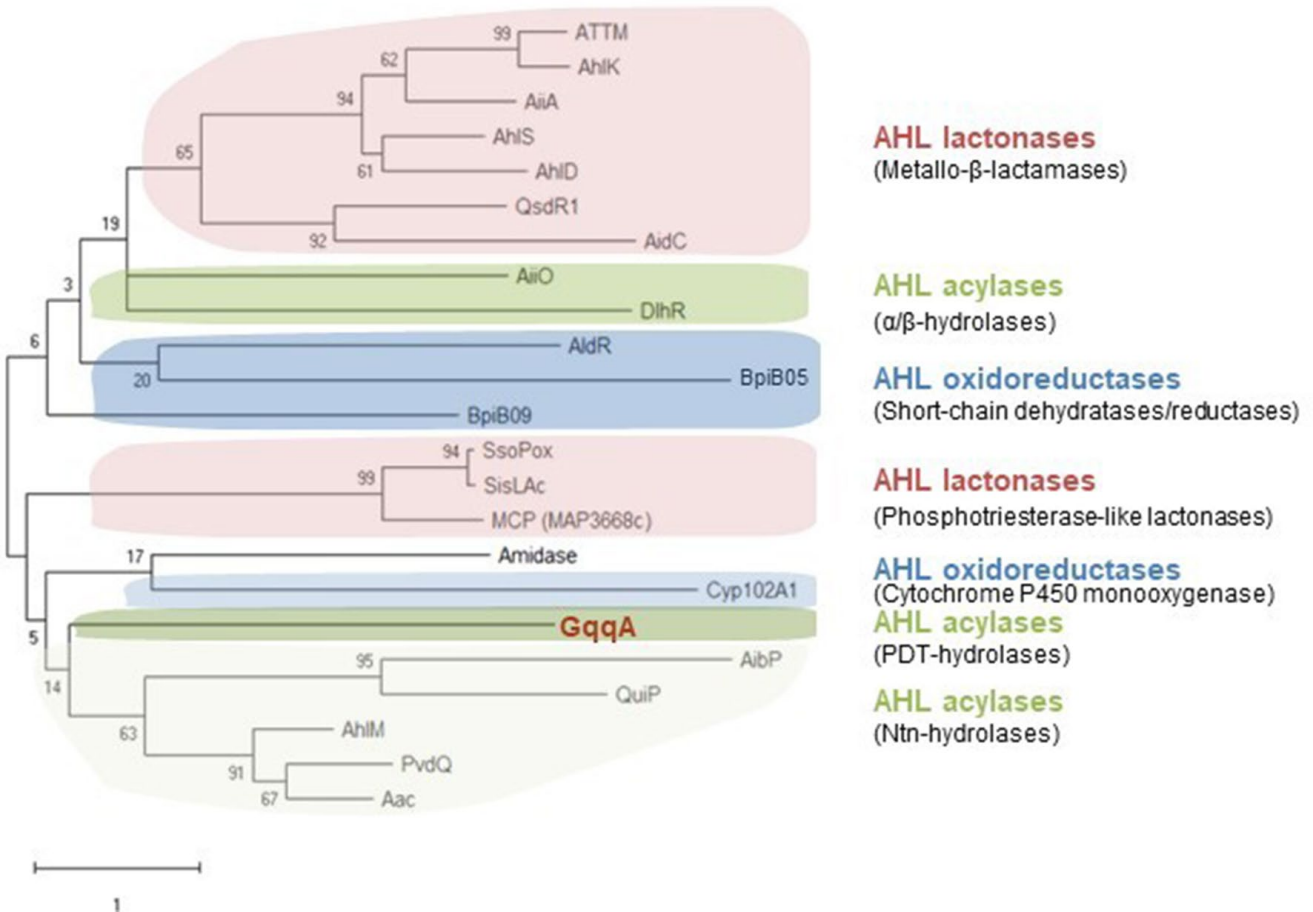
Phylogenetic diversity of known QQ enzymes acting on AHLs. The evolutionary history was inferred by using the Maximum Likelihood method and JTT matrix-based model 52 53. The tree with the highest log likelihood is shown. The percentage of trees in which the associated taxa clustered together is shown next to the branches. Initial tree(s) for the heuristic search were obtained automatically by applying Neighbor-Join and BioNJ algorithms to a matrix of pairwise distances estimated using a JTT model, and then selecting the topology with superior log likelihood value. The tree is drawn to scale, with branch lengths measured in the number of substitutions per site. This analysis involved 23 amino acid sequences. There were a total of 1049 positions in the final dataset. The different enzyme classes are highlighted in different colours and many enzymes were chosen 10. The enzymes depicted are: PvdQ (Q9I194), Aac (Q8XWC7), AhlM (AAT68473.1), AibP (Q8YDG4), QuiP (Q9I4U2), GqqA (KOEU_05990), Amidase (Q396E7), Cyp102A1 (P14779), MCP (MAP_3668c), SsoPox (Q97VT7), SisLAc (C4KKZ9), AiiO (D8X182), DlhR (ACP22150.1), QsdR1 (ACP23138.1), AidC (I7HB71), AhlS (SCV20999.1), AhlD (AAP57766.1), AiiA (P0CJ63), AttM (Q7D3U0.3), AhlK (A0A098CCK3), BpiB09 (ABU51100.1), AldR (ACP26074.1) and BpiB05 (ABU51109.1). Corresponding accession numbers are given in brackets after enzyme name.

Where signals are produced the destruction is one way of quenching the message. In nature signals are usually degraded over time and today we know that several bacterial enzymes exist that are involved in breakdown of AHL molecules. These enzymes are collectively called Quorum Quenching (QQ) enzymes and are summarized in recent reviews^10–13^. Today over 30 in part characterized enzymes are known acting on AHLs and derived from a wide range of different bacteria and also a number of non-cultivated species. Figure 1 summarizes the current phylogenetic diversity of known and biochemically characterized QQ enzymes.

Three main types of enzymes are known to act on AHLs. These are lactonases (EC. 3.1.1.-), acylases (EC 3.5.1.–) and oxidoreductases (EC 1.1.–) (Fig. 1). For each of the main enzyme classes two subclasses of enzymes are known. While a few oxidoreductases have been reported the best studied group of enzymes processing AHL molecules are hydrolases, including lactonases and acylases^14–16^. Thereby lactonases hydrolyze the lactone ring in a reversible way. One of the first QQ enzymes reported was the lactonase AiiB from *Bacillus subtilis*^17^.

Acylases (amido-hydrolases) hydrolyze the amide group and release the homoserine lactone and the carboxylic acid. The currently known acylases separate in two distinct groups: The N-terminal nucleophile aminohydrolases (Ntn hydrolases) and the α/β-hydrolases (Fig. 1). Ntn hydrolases cleave an amide bond^18,19^. The N-terminal fold further provides the possibility of autocatalytic processing^19^. The majority of the currently known AHL acting acylases belongs to the Ntn hydrolases^11,20–31^. Today only the *Ochrobacterium* sp. A44 AiiO and the *Sinorhizobium* NGR234 DlhR appear to be α/β-hydrolase-like acylases^30^. The α/β-hydrolases are an extended group of very diverse but structurally conserved enzymes. Recently, we published a newly identified QQ enzyme designated GqqA^1^. The *gqqA* gene encodes for a protein with 281 amino acid residues and a corresponding molecular mass of 30 kDa. The polypeptide sequence lacks the typical features of both, the Ntn hydrolase and the α/β-hydrolase. However, the sequence of the protein shows high similarity to predicted prephenate dehydratases (PDTs; EC 4.2.1.51), which are involved in the metabolic pathways of aromatic amino acids.

**Figure 2:**
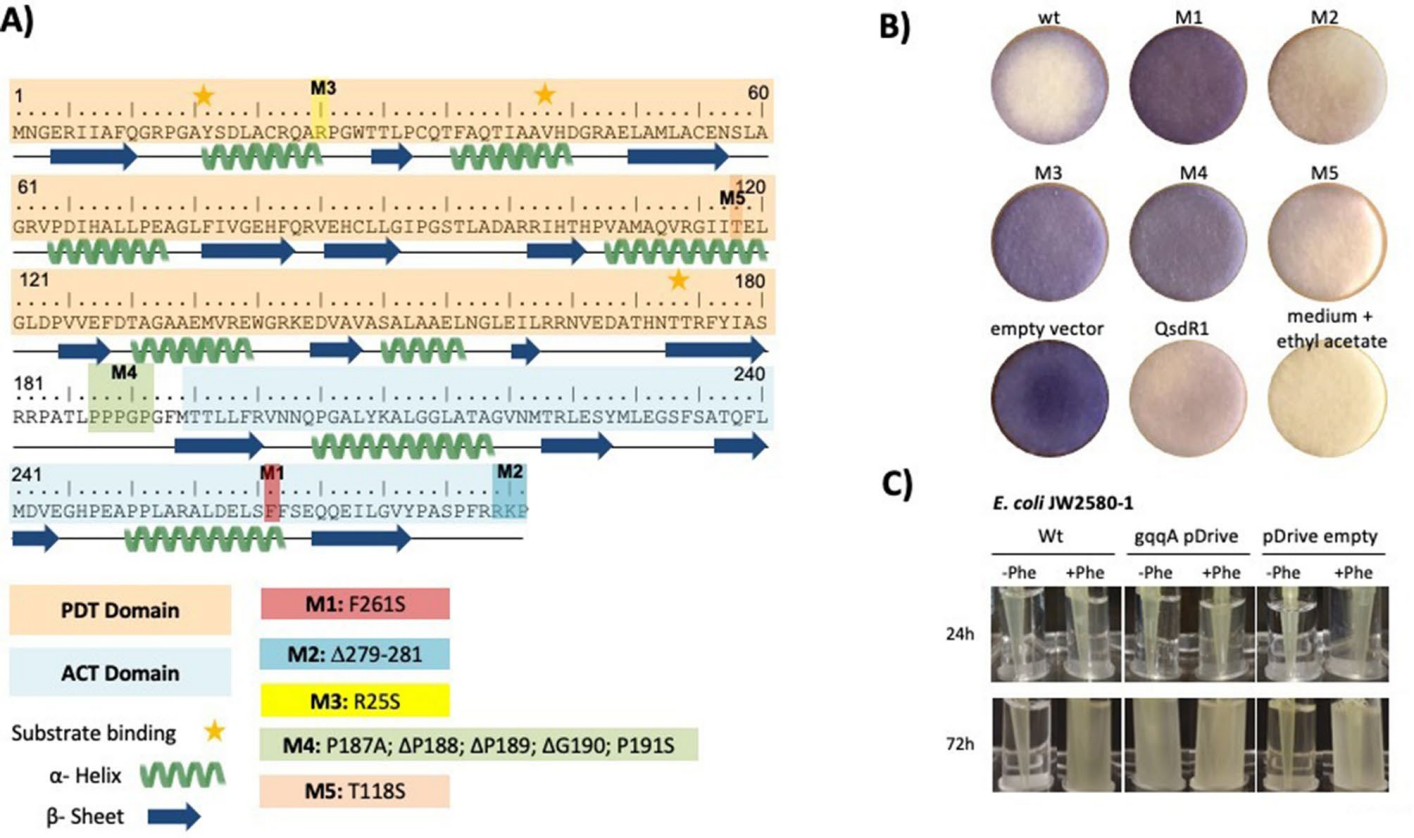
(**A**) Secondary structure of GqqA and (**B**) bioactivties of GqqA. The aa sequence of GqqA with the associated secondary structure is shown. The PDT domain is colored in orange and the ACT domain in light blue. The aa changes in the mutants (M1-M5) are highlighted in different colors. M1 (F261S) was shown in red, M2 (ΔRKP) in blue and M3 (R25S) in yellow. The modification at the loop (M 4) was highlighted in green and the aa exchange T118V (M5) in orange. (**B**) Bioindicator assay of an in vivo enzyme assay of GqqA wt and various mutants in *E. coli*. The reporter strain CV026 was used for indicating not degraded AI molecules (purple color)^52^. An active enzyme is shown by a colorless plate. An *E. coli* strain harboring the empty expression vector pET-21a was used as negative control. The quenching enzyme QsdR1 served as a positive control^30^. (**C**) Genetic complementation assay using the phenylalanine auxothrophic *E. coli* JW2580. WT, cultures of *E. coli* 2580–1 without plasmid; gqqA pDrive, 2580–1 containing gqqA in pDrive and pDrive empty; 2580–1 with the empty pDrive vector as additional control. Strains were grown in LB medium at 37 °C over-night prior to the tests. The cells were harvested and washed three times in M9 medium. 5 ml M9 medium with or without phenylalanine (2 µM) were inoculated with 50 µl of the washed cells and incubated up to 72 h at 37 °C. Images show representative cultures of at least three independent complementation tests.

Prephenate dehydratases are part of the phenylalanine biosynthesis pathway and catalyze the decarboxylation of prephenate to phenylpyruvate. In some bacteria, such as *Escherichia coli*, the PDT is part of a relatively large bifunctional P-enzyme that also has a chorismate mutase function (CM, EC 5.4.99.5). Thereby the amino acid residues 1–109 harbor the CM function and residues 101–285 the PDT activity. In addition, the structural region of amino-acid residues 286–386 contains an independent regulatory-domain (ACT-domain) that is involved in a regulatory feedback system by coordinating L-Phenylanalnine^32,33^. *Komagataeibacter europaeus CECT 85,646* codes in its 4.1 Mb genome for a single predicted PDT, designated GqqA^34^. GqqA has a length of 281 amino-acid residues and shares 31% identity to the *E. coli* PDT enzyme. However, the *K. europaeus N-terminal aa of* GqqA lack homology to the chorismate mutase domain but contains the C-terminal regulatory domain, indicating that the chorismate mutase activity is encoded as a separate protein (Ga0098768_10078, tyrA1) elsewhere in the genome^34^ (Fig. 2A). GqqA overexpression in the host strain *Komagataeibacter europaeus* interfered with QS-dependent biofilm and cellulose formation and further experiments provided strong evidence that GqqA wildtype protein actively quenches AHL signaling molecules^1^ (Fig. 2B)*.* Intrigued by the observation that GqqA has a rather low homology to so far known QQ enzymes, however, is sharing a high similarity with PDT enzymes, we were interested to analyze its function in terms of AHL modification. Therefore, we assayed the hydrolysis of AHLs with recombinant GqqA and applied ESI–MS–MS to identify the reaction products. We solved and refined the native GqqA three-dimensional structure to 2.5 Å resolution, utilizing SAD phases obtained from a truncated and Se modified version of GqqA. Structural data together with a phylogenetic analysis and the enzymatic characteristics implied that GqqA represents the first example of a till now unknown, third and novel subclass of acylases. They act on AHLs and modern ß-lactam antibiotics, thus adding a new and complementary functionality to the PDT family of enzymes.

## Results and discussion

### Recombinant GqqA is a multifunctional enzyme acting as an acylase on AHLs, ß-lactam antibiotics and complements E. coli pheA phenotype

Previously, we showed that GqqA actively quenches AHL signalling molecules using reporter strains^1^. GqqA is a 281 aa protein consisting of eight α-helices and 15 β-sheets (Fig. 2A). The protein reveals two major functional domains. The predicted PDT domain stretches from position 1–180 and the ACT regulatory domain stretches from aa position 194–281 (Fig. 2A). To further characterize the protein GqqA was heterologously overproduced and purified similar to the previously published protocol^1^. The purified protein was verified by SDS-PAGE and Western blotting with associated immune detection of the His-tagged GqqA. Purified GqqA produced a single band on SDS-PAGEs corresponding to approx. 30.5 kDa (Figure S1, A), which is in accordance with the calculated molecular mass. The SDS-PAGEs indicated that the protein was homogenous with only very minor contaminations. Experiments to access the activity, using initially either *Chromobacterium violaceum* CV026 and/or *Agrobacterium tumefaciens* NTL4 as reporter strains and 3-oxo-C8-HSL as substrate, confirmed that the purified protein was active (Fig. 2B).

The active protein was incubated with oxo-octanoyl homoserine lactone (3-oxo-C8-HSL) as substrate and the enzymatic cleavage products were analyzed using ESI–MS–MS. The employed oxo-C8-HSL has a molecular mass of 241.26 g/mol. After incubation of oxo-C8-HSL with recombinant GqqA we observed a molecular mass of 134.08 in the ESI positive mode and a molecular mass of 157.09 in the ESI negative mode. The 134.08 peak [M + MeOH + H]^+^ corresponded to the homoserine lactone with a MW of 101.05 g/mol and the 158.09 [M–H]^−^ peak corresponded to 3-oxo-octanoic acid (MW of 158.09 g/mol). Further analyses implied that GqqA can transforms the four different 3-oxo-acyl homoserine lactones tested, with 3-oxo-decanoyl homoserine lactone being the preferred substrate (Table 1). Our biochemical assays implied that GqqA had a V_max_ of 152 ± 6.3 U g^−1^ with oxo-C10–HSL and a-V_max_ of 117.3 ± 12.8 using 3-oxo-C8-HSL (Table 1). This is in agreement with the structure analysis which suggests that the active site of GqqA allows to cover fatty acid residues with variable lengths. These data are also in line with our previous results using reporter strains^1^.

**Table 1:** Kinetic data for the conversion of 3-oxo-acyl homoserine lactones by GqqA.

Substrate	*V*_max_ (units × mg^−1^)	K_m_ (mM)^a^	*V*_max_/Km (units × mg^−1^ mM^−1^)
3-Oxo-hexanoyl homoserine lactone	109.3 ± 5.1	6.89 ± 0.15	15.86
3-Oxo-octanoyl homoserine lactone	117.3 ± 12.8	4.87 ± 0.12	24.08
3-Oxo-decanoyl homoserine lactone	152.3 ± 6.3	2.36 ± 0.35	64.53
3-Oxo-dodecanoyl homoserine lactone	13.77 ± 8.17	16.7 ± 0.5	0.82

^a^For the Michaelis–Menten fits see Figure S2.

**Figure 3:**
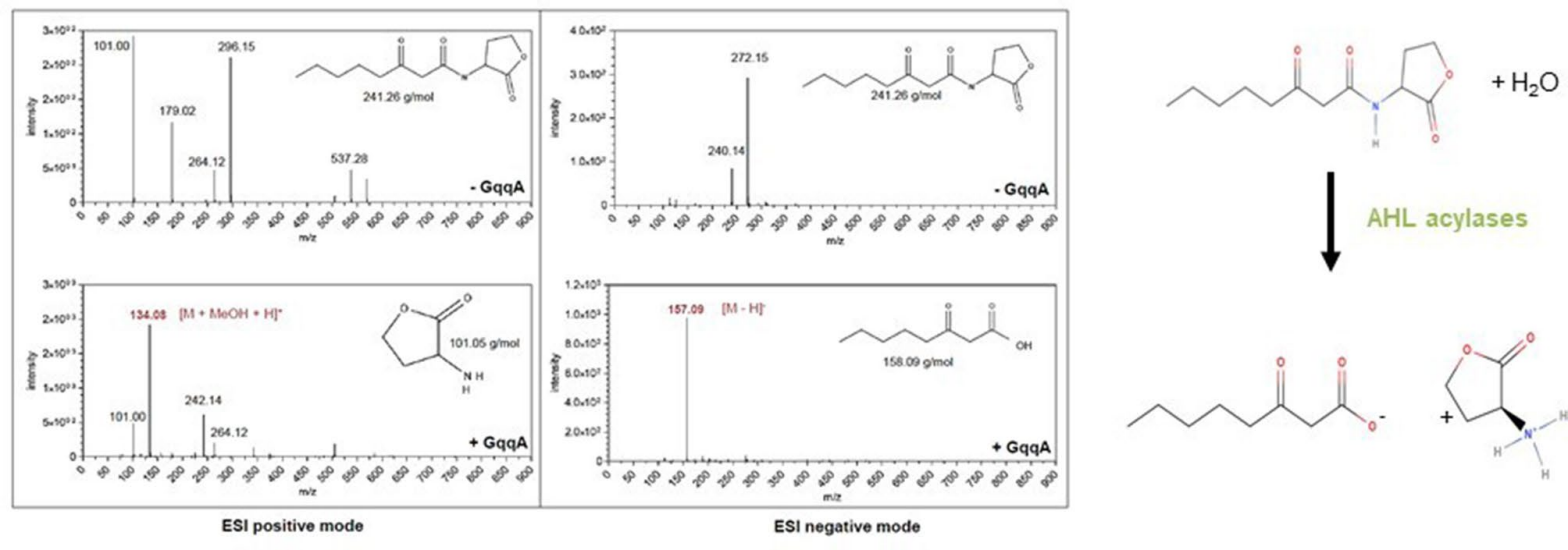
ESI/MS measurements of GqqA on 3oxo-octanoyl homoserine lactone. The upper spectra indicate the control without enzyme (-GqqA) in the positive (left) and in the negative (right) mode. MeOH was used as solvent during MS analysis, it appears an esterified substrate or product. The control spectrum in the positive mode shows an [M + MeOH + Na]+ ion at an m/z of 264.14 and an [2 M + 2MeOH + Na]+ ion at m/z of 537.28 that indicate the substrate 3oxo-octanoyl homoserine lactone. The structural formula is drawn in the spectrum. The substrate is also observed at an m/z of 240.14 [M–H]—in the negative mode. By adding GqqA to the assay (lower spectra) the substrate is only observed in lower intensity. An [M + MeOH + H] + ion is observed at an m/z of 134.08 in the positive mode and indicates the product homoserine lactone. In the negative mode an [M–H]—ion is detectable at an m/z of 157.09 and displays the 3- oxo- octanoyl acid. The homoserine lactone and the 3-oxo- octanoyl acid are displayed in the spectra. The [M + Na] + ion at an m/z 101.00 and the [2 M + Na] + at an m/z at 179.02 display.

These observations implied that GqqA acts as an acylase by hydrolyzing the acyl substituent of the lactone ring, but not as lactonase (Fig. 3). Further biochemical assays implied that GqqA had a V_max_ ranging from ca. 14 U mg^−^1 for 3-Oxo-dodecanoyl homoserine lactone to ca. 152 U mg^−1^ for 3-Oxo-decanoyl homoserine lactone, which were found as the less and best preferred substrate according to V_max_/K_m_ values (see Table 1 and Figure S2).

Further we asked to which extend GqqA would be promiscuous with respect to the substrate profile. To answer this question, we investigated the degradation of various ß-lactam antibiotics by GqqA. In a disc diffusion antibiotic susceptibility test, ß-lactam antibiotics were used and the effect after incubation with GqqA on *S. aureus* cells was tested. These assays demonstrated that GqqA effectively degrades Penicillin G, Amoxicillin and Ampicillin (Figure S3). However, it was not active against the Cefotaxime. The latter is in contrast to the other ß-lactam antibiotics tested a 3^rd^ generation ß-lactam antibiotic and structurally more advanced. This explains why Penicillin G, Amoxicillin and Ampicillin were cleaved. Altogether our data imply that GqqA reveals at least some promiscuity. Within this framework, it is notably, that the recently uncovered acylase MacQ has been described to be involved in QQ and antibiotic resistance. Thereby, MacQ acts against AHLs and Penicillin G and is an acylase belonging to the Ntn family^21^.

Since GqqA revealed surprisingly high similarities to prephenate dehydratases, we also asked if an *E. coil pheA* mutant could be complemented by *gqqA*. In the complementation assays we used the phenylalanine auxotrophic *E. coli* JW2580-1. This mutant carries a kanamycin gene inserted into *pheA* and is thus auxotrophic for phenylalanine (TABLE S1). Notably, growth of this strain could be restored in phenylalanine deficiency medium after introducing *gqqA* on the self-replicating plasmid pDrive. Cells carrying extra copies of *gqqA* usually grew within 24 h to optical densities of OD 600 nm 0.5 (Fig. 2C). Clearly, these findings imply that GqqA retains its prephenate dehydratase activities.

The observation here that GqqA has an additional acylase activity may explain some of the earlier observations that have been observed for *pheA* mutations in other Gram-negative bacteria but that could not be explained by the simple knockout of an amino acid biosynthesis pathway. Among those the recent observations that motility and pathogenicity, which are usually QS-dependent processes in *Acidovorax citrulli*^35^ were altered in the background of a *pheA* mutant. Further the increased persistence of the opportunistic pathogen *Pseudomonas aeruginosa* had been reported in a *phe* mutant and may also hint into a similar direction^36^.

### DLS and SAXS experiments

**Figure 4:**
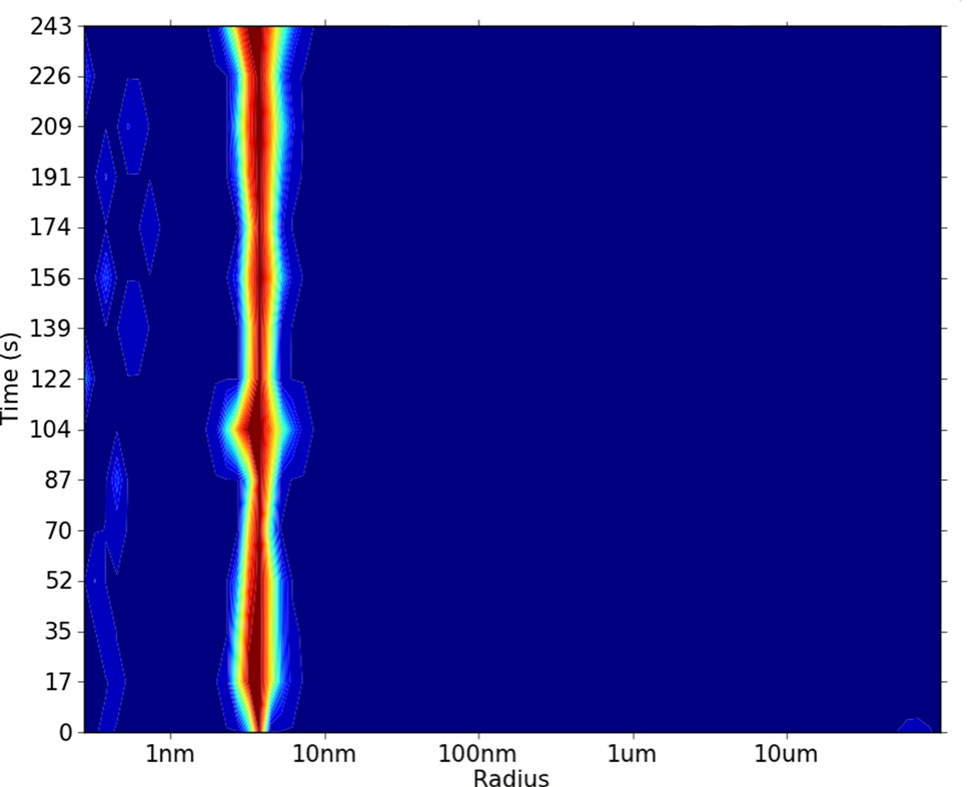
DLS Measurement of recombinant and purified GqqA. DLS measurements of GqqA (3.6 mg ml^−1^) revealed a hydrodynamic radius of 3.9 ± 0.1 nm, corresponding to a GqqA dimer (approx. 61 kDa). The relative abundancy of particles is color-coded from dark blue (low) to dark red (high).

The homogeneity of recombinant GqqA was analyzed via DLS measurements at 20 °C. A single peak was monitored corresponding to a hydrodynamic radius of 3.9 ± 0.1 nm, which is indicating a GqqA dimer of 61 kDa in solution (Fig. 4). Long-term DLS measurements at room temperature showed that the GqqA dimer remains stable for at least 4 days (Figure S4, A). Also, data obtained from SAXS experiments verified a GqqA dimer in solution. SAXS data revealed a radius of gyration of 2.7 nm and a d_max_ of 7.56 nm (Table S4).

### Crystal structure analysis of GqqA

Crystals of GqqA and SeGqqA grew applying seeding within 3 days and belong to the space group *P*2_1_ (GqqA) and *P*2_1_2_1_2_1_ (SeGqqA), respectively (Figure S4 B, C)_._ Molecular replacement calculations using the diffraction data of native GqqA collected to 2.5 Å resolution and the most homologous protein structure deposited at pdb at that time, a prephenate dehydratase with 33% sequence identity (pdb code 2qmx), was not successful to solve the phase problem. Therefore, the crystal structure of SeGqqA was solved by single-wavelength anomalous diffraction (SAD) and refined to 1.7 Å resolution. The coordinates of the solved and refined structure of SeGqqA were suitable to solve the phase problem of native GqqA data by molecular replacement.

**Figure 5:**
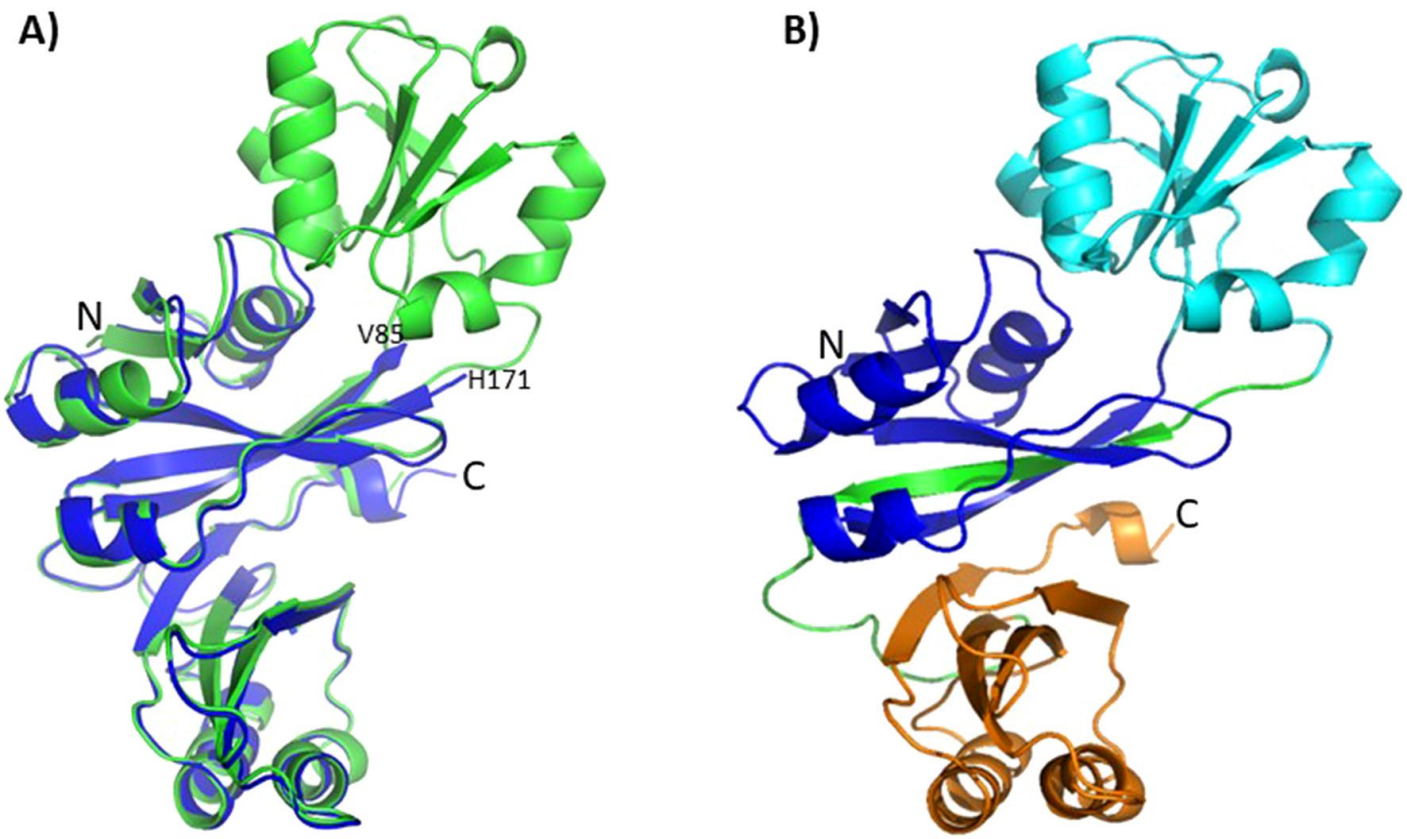
Structural analysis of GqqA and SeGqqA. (**A**) superposition of GqqA (green) and SeGqqA (blue) highlights that one protein domain is missing in SeGqqA. The artificial termini after truncation are labelled (V85, His171). (**B**) The N-terminal (blue) and C-terminal (orange) domains are stabilized through a linker (green), which connects the middle domain to the C-terminal ACT domain. This linker is integrated in the N-terminal β-sheet.

GqqA consists of three subdomains. Upon structure solution and refinement of SeGqqA we realized that one subdomain (residues 86–170) is missing, shown and indicated in Figure 5A. The loss of this domain, which obviously appeared by proteolysis during the crystallization process of SeGqqA, resulted in crystals with different space group and cell dimensions, compared to GqqA. However, the obtained crystals were of higher quality. Due to the fold of the polypeptide chain in GqqA the missing domain for SeGqqA is located entirely at one side of the protein and has only a few interactions with the other domains (Figure 5B). Superposition of the native GqqA on SeGqqA main chain atoms resulted in a Cα rms deviation of 0.76 Å, indicating that the missing domain of the SeGqqA structure did not change the overall fold of remaining two protein domains.

**Figure 6:**
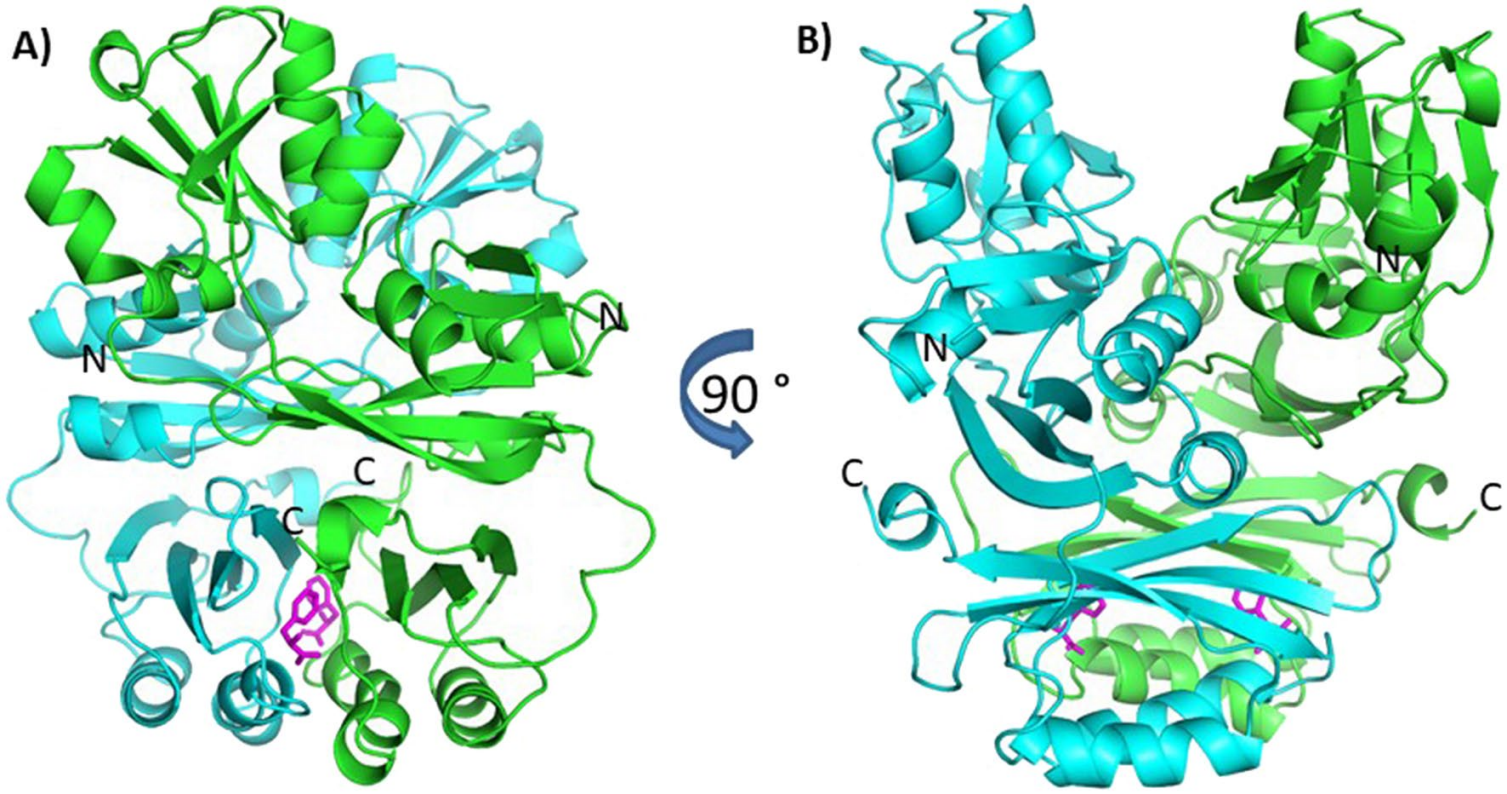
Homodimer of GqqA with labelled N- and C-termini. Monomers are shown in cyan and green. Within the C-terminal ACT domain a four-stranded anti-parallel β-sheet of one monomer with its symmetry related mate complement an extended β-sheet structure. In the dimer interface a L-Phe molecule (pink) is bound in both monomers.

DLS and SAXS measurements indicated a GqqA dimer in solution, which is also present in the crystal structure with a rather extended interface area of approx. 2230 Å^2^ between the monomers (Figure 6). A GqqA monomer itself consists of three domains. Two of them, the N-terminal and middle domain form the functional unit, which is homologous to the catalytic PBP domain of the prephenate dehydratase (pdb code: 2qmx), whereas the C-terminal domain is homologous to the regulatory ACT domain. The N-terminal and middle domain of GqqA consist of a mixed five-stranded β-sheet with three parallel strands, surrounded by three α-helices. The C-terminal ACT domain is formed by a four-stranded anti-parallel β-sheet and two helices. The symmetry related mate of the ACT domain complements an extended central β-sheet structure of the functional homodimer (Figure 6). In the C-terminal regions of both monomers within the dimer interface we identified two L-Phe molecules. Bound L-Phe molecules were also identified in the *E. coli* prephenate dehydratase structure (pdb code 2qmx), where they are assigned to be responsible for feedback regulation^37^, triggering allosteric events by changing the relative orientation of the ACT-domains in the homodimer. L-Phe is also bound in the truncated SeGqqA.

### Comparison of GqqA and the homologous prephenate dehydratase

**Figure 7:**
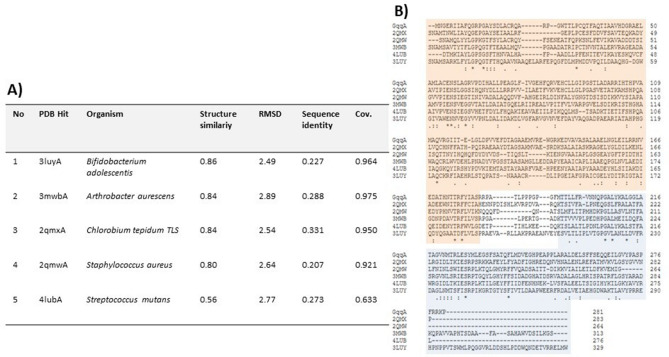
A GqqA alignment with related proteins of different origins. Prephenate dehydratases show the highest homology to GqqA. However, the alignment with different PDTs (PDB codes: 2QMX, 2QMW, 3MWB, 4LUB, 3LUY) reveal still low sequence identity, meanwhile the structural similarity is very high.

**Figure 8:**
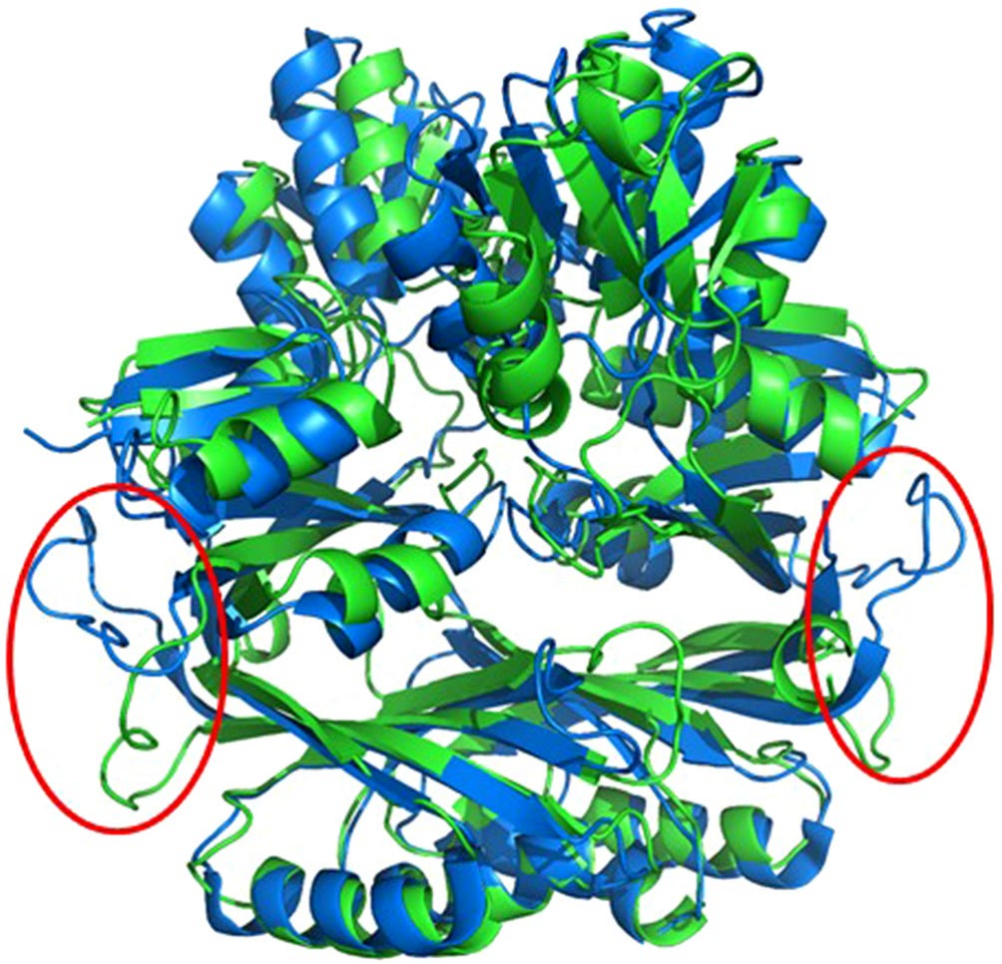
Superposition of GqqA and the prephenate dehydratase (2qmx). The structures of GqqA (green) and 2qmx (blue) share the same fold but the domains are in a different orientation to each other. The main difference is located at the loop between both functional units (red circles) which have completely different sequences and orientation.

Overall, the structure of GqqA is highly homologous to known structures of PDT enzymes. A DALI^38^ search identified the highest structural similarity of GqqA and a PDT from *Bifidobacterium adolescentis* (PDB: 3luy), with a sequence identity of 22.7% and C_α_ rms value of 2.5 Å (Figure 7A). A sequence comparison of GqqA with various prephenate dehydratases showed that the PBP domain is more conserved than the ACT domain (Figure 7B). Superposition with the prephenate dehydratase structure of *Chlorobium tepidum* TLS (2qmx) which shares 33% sequence identity and rmsd of 2.3 Å on Cα-atoms, revealed that the catalytic and the regulatory domains of the proteins share the same fold, but the catalytic domains have slightly different orientations to each other. In GqqA the loop formed by residues 181–192 is slightly shorter than the corresponding loop of the *Chlorobium tepidum* TLS prephenate dehydratase (PDB: 2qmx, residues 178–192) and has a different conformation (Figure 8).

### The active site of GqqA

Crystal structures of GqqA closely related enzymes with bound substrates or inhibitors are not published. Our efforts to get substrate or inhibitor complexes of GqqA by co-crystallization with N-acyl homoserine lactones or soaking native crystals with substrates or inhibitors failed. The electron density maps of several diffraction datasets revealed no electron density to localize a putative active site.

The PBP domain is folded into two structurally similar subdomains which form a cleft for the putative active site. Regarding other structures which share PBP domains, we suggest an active site in a cleft between the subdomains of the catalytic PBP domain. The DALI server^38^ was used to find the most homologous structure with a bound substrate. An ATP phosphoribosyltransferase (pdb code: 1nh8) shares 12% sequence identity with GqqA and a rmsd of 4.3 Å on Cα-atoms. This protein has an AMP bound in the above-mentioned cleft of the PBP domain^39^. GqqA possess a highly hydrophobic cleft between these domains which gave a suitable environment for the hydrophobic part of N-AHLs.

**Figure 9:**
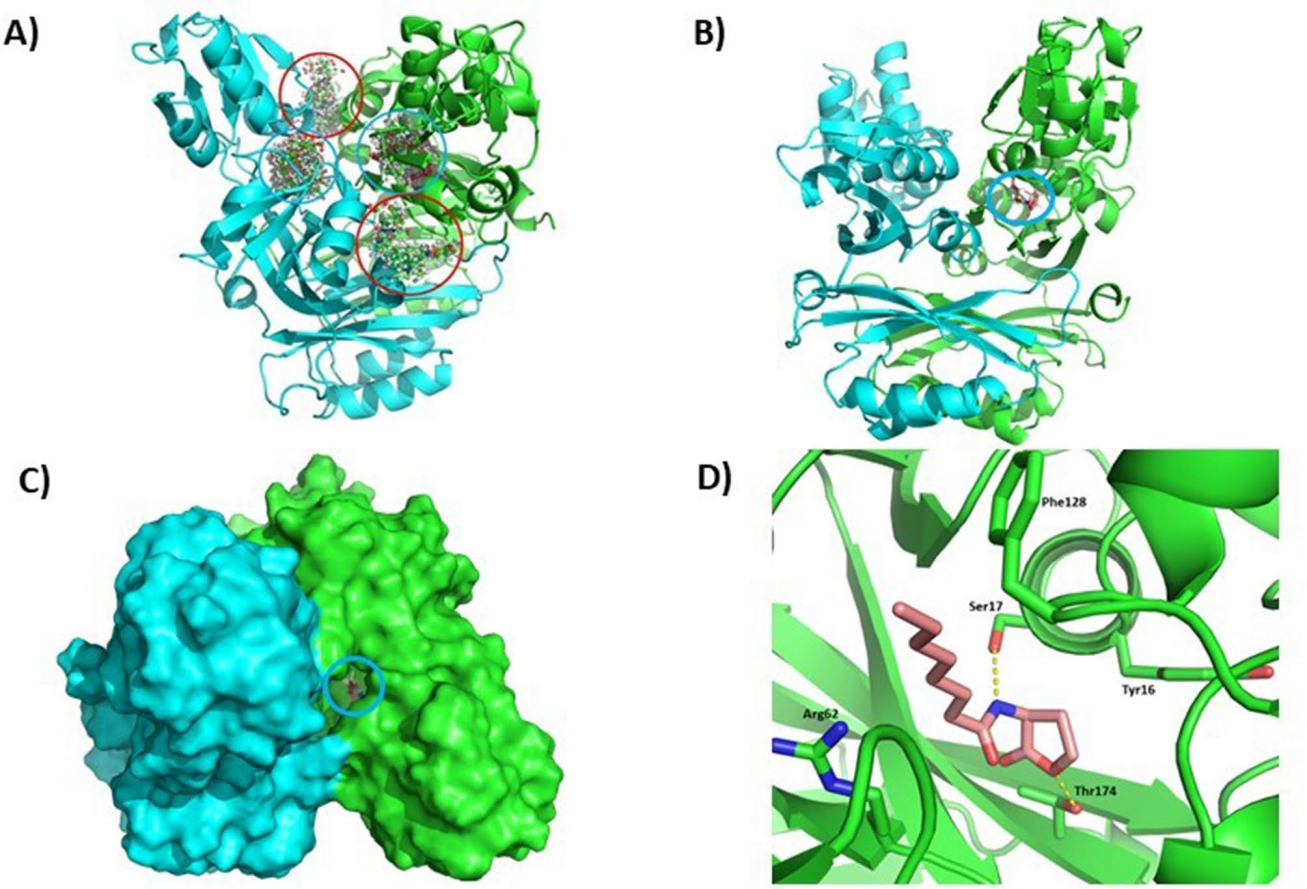
Identification of a putative active site of GqqA by docking studies with N-octanoyl-L-homoserine lactone. (**A**) SwissDock revealed clusters of two possible binding sites for each monomer (red circles or blue circles). The putative active site is located in the cleft between the subdomains of the catalytic PBP domain (blue circles). Another binding site is proposed on the solvent exposed surface in the neighbourhood of the active site (red circles). (**B**–**D**) Example of a possible ligand docking (blue circles in **B, C**); Thr174 is in our model located at the bottom of the cleft near the protein interface. The modelled ligand (N-octanoyl-L-homoserine lactone) is most likely positioned between Ser17 and Thr174 and as proposed in (**D**). Thr174 as well as Ser17 are able to build hydrogen bonds to the ligand indicated with dots. Tyr16, Arg62 and Phe128 interact via van-der-Waals forces.

Docking studies applying the native GqqA structure and the potential substrate N-octanoyl-L-homoserine lactone suggest that the lactone ring most probably binds to the polar residues Y16, S17 and T174 located in the cleft typical for the subdomains of PDT (Figure 9). A minor binding site of the ligand has been identified on the surface of GqqA (Figure 9A). The polarity of the cleft is enhanced by the helical dipoles of three α-helices pointing with their N-termini into the cleft. Also, the C-termini of the parallel β-sheet of the catalytic domain point towards the cleft. The nonpolar alkyl chain of AHL is directed to the entrance of the active site of GqqA, allowing to cover fatty acid residues with variable lengths.

For prephenate dehydratases structures an open and a closed conformation has been observed, which is regulated by phenylalanine binding^37^. In the open conformation the putative active site region is more solvent accessible. According to this data the GqqA crystal structure we analysed corresponds to a closed conformation.

Overall, the structural homology of GqqA with structures of PDTs implies that GqqA evolved from a prephenate dehydratase and the substrate specificity was enhanced, due to mutations in its active site region. In the *E. coli* prephenate dehydratases four amino acid residues were identified to be important for prephenate dehydratase catalytic activities^32,33^. However only two were conserved in GqqA (N206 and L215). Further in *E. coli* the two amino acids E159 and E232 appear to be important for substrate binding^33^. These two amino acid positions are conserved in GqqA and correspond to E55 and E127.

Notably, the above-described complementation studies (Figure 2C) strongly imply that GqqA has retained its native prephenate dehydratase activities. Growth of the *E. coli* PDT mutant would not be possible without a functional PDT activity of GqqA.

### Analysis of different mutations of GqqA

To obtain functional insights about the dimerization we analyzed the dimer interface region and identified essential interactions and amino-acid residues stabilizing the dimer. To address these regions, five GqqA mutants (Figure 2) were generated. The correctness of the different mutations was verified by sequencing and the constructs were transformed into the overexpression strain *E. coli* BL21 (DE3), purified and assayed for their function (Figure 2B).

In GqqA_F261S (M1), this mutation probably reduced essential hydrophobic interaction within the dimer interface and as a result GqqA_F261S (M1) was inactive, when assayed with the *Chromobacterium violaceum* reporter strain CV026 (Figure 2B). The second mutation (GqqA_Δ279-281) (M2) truncated the C-terminus without effects on the enzymatic activity. The GqqA_R25S (M3) mutant affected the dimerization resulting again in a loss of activity (Figure 2B). A fourth mutation (GqqA_P187A_ΔP188_ΔP189-190_P191S) (M4) was introduced with the goal to remove the most divergent loop, at residues 187–191. This mutant was not active. A fifth mutation (M5) was introduced and targeted the threonine at position 118 and which was changed to a valine. This mutant was active implying that the threonine 118 was not involved in the catalysis.

## Conclusions

Earlier we have identified GqqA in the cellulose overproducer and biofilm-forming bacterium *K. europaeus* CECT 8546, interfering with QS signals of AHLs from Gram-negative bacteria^1,34^. GqqA acts as an acylase on AHL and the amido hydrolyzing mechanism is unambiguously characterized by ESI–MS/MS analysis, identifying homoserine lactone and the fatty acid as products. The crystal structure confirmed that GqqA can be assigned to be a first member of a novel type of acylases, since GqqA has no homology to currently known Ntn hydrolases or alpha/beta hydrolases. Instead, the structure has a high homology to prephenate dehydratases, indicating that GqqA developed from this class of enzymes, which are all active as dimers. While the mutational analyses in combination with the structural data gave a first insights into the function of this remarkable and bifunctional enzyme future research will have to address additional questions. Thereby, the most immediate steps will be a mutational verification of the substrate binding site and gaining a better understanding on the mechanisms involved in substrate specificity versus promiscuity.

## Materials and methods

### Bacterial strains, plasmids, primers and growth conditions

Bacterial strains and plasmids utilized in terms of the investigations are summarized in Table S1. *Escherichia coli* strains were cultivated in LB or M9 medium at 37 °C. Antibiotics were added to the cultures, when required, with appropriate concentrations.

### Overexpression and purification of GqqA

The gene encoding GqqA, isolated from *Komagataeibacter europeaus*, was integrated in pET21a with a C-terminal TEV-protease cleavage site (ENLYFQS) and a His_6_-tag, as described before by^1^. GqqA was produced by gene overexpression in *E. coli* strain BL21 (DE3). The culture was grown in LB medium with ampicillin (final concentration 100 µg ml^−1^) inoculated with 25 ml pre-culture at 37 °C. When the cells reached the exponential growth phase (OD_600nm_), protein expression was induced by supplementation of 1 mM IPTG (isopropyl-β-D-1-thiogalactopyranoside). After 3 h of incubation the cells were harvested by centrifugation applying 4000* g* at 277 K for 30 min. The pellet was resuspended in lysis buffer (50 mM NaH_2_PO_4_, 300 mM NaCl, 10 mM imidazole, pH 8.0). The cells were disrupted by 5 × 1 min sonification and centrifuged with 17000* g* at 277 K for 1 h. The supernatant containing the His_6_-tagged target protein was incubated on a Ni–NTA agarose column for 1 h on a rotary shaker at 277 K. The sample was washed twice with wash buffer (50 mM NaH_2_PO_4_, 300 mM NaCl, 20 mM imidazole, pH 8.0) and eluted with 5 ml elution buffer (50 mM NaH_2_PO_4_, 300 mM NaCl, 250 mM imidazole, pH 8.0). The sample was concentrated by centrifugation applying amicon filters and loaded onto a size-exclusion chromatography column (Superose6Increase, GE Healthcare) in a buffer consisting of 0.1 M K_2_HPO_4_, pH 5.0, 150 mM NaCl. Fractions containing the pure protein were pooled and concentrated to 10 mg ml^−1^. The purity of the protein was verified by Coomassie stained SDS-PAGE (FIGURE S1 A), which shows a dominant band at approximately 30 kDa, consistent with monomeric GqqA (30.5 kDa).

To solve the crystallographic phase problem the 8 methionines of GqqA were substituted by selenomethionines^40^. The preculture was centrifuged with 17,000* g* at 4 °C for 20 min. and resuspended in 5 ml M9-medium. The culture was grown in M9-medium with Ampicillin (final concentration 100 µg ml^−1^) inoculated with 1.25 ml resuspended pellet at 37 °C. When the cells reached the exponential growth phase (OD_600nm_), they were supplemented with 20 ml selenomethionine (SeMet) (1 g l^−1^) and 5 ml of an amino acid mixture (TABLE S2). After 15 min protein expression was induced by supplementation of 1 mM Isopropyl-β-D-thiogalactopyranoside (IPTG) and after 3 h of incubation at 37 °C the cells were harvested by centrifugation with 4,000* g* at 4 °C for 30 min. The selenomethionine-variant of GqqA (SeGqqA) was purified following the same procedures as applied for GqqA.

### ESI–MS/MS analysis

For enzyme assays 3-oxo-octanoyl homoserine lactone (oxo-C8-HSL) as substrate was used in 5 mM N-2-hydroxyethylpiperazine-N’-3-propanesulfonic acid (EPPS) buffer, pH 8.0. Prior to use a 100 mg/ml stock solution of oxo-C8-HSL in dimethyl sulfoxide (DMSO) was prepared. If otherwise not indicated, reactions were performed, in triplicates and corrected for non-enzymatic transformation, at—70° and in 5 mM EPPS buffer, pH 8.0. The assays were conducted according to the following steps. One milliliter of 5 mM EPPS buffer, pH 8.0, was introduced into a 1.5 ml Eppendorf tube that was closed tightly. The substrate oxo-C8-HSL was then added to a final concentration of 0.5 mg/ml (from a 100 mg/ml stock solution in DMSO). Finally, the protein was immediately added to a final concentration of 10 µg/ml (from a stock solution of 5 mg/ml in 40 mM HEPES, pH 7.0). Aliquots were taken at different time intervals, and the reaction products were analyzed by Mass Spectrometry (MS) experiments, performed in a hybrid quadrupole-time of flight mass spectrometer (QTOF, QSTAR pulsar i, ABSciex) equipped with a micro electrospray ion source (in positive and negative mode). Samples were dissolved in methanol (0.1 ml sample plus 0.9 ml methanol) and introduced in the spectrometer using a syringe infusion pump with a 10 ul/min flow. N_2_ was employed in the collision cell. MS experiments in TOFMS mode were registered to identify the reaction products. The experiments were performed with positive ion detection. The instrumental parameters were set as follows: mass range 50–2000 Dalton; ion spray voltage (IS): 4500 V; ion source gas pressure (GS1): 10 psi; curtain gas pressure (Cur): 20 psi; declustering potential (DP): 30 V; focusing potential (FP): 210 V; declustering potential 2 (DP2): 15 V; collision gas: 3. Substrates and products used and generated are listed in TABLE S3 together with their molecular mass.

### Dynamic light scattering measurements

Prior to SAXS and crystallization experiments the homogeneity of GqqA and SeGqqA in solution (3.6 mg ml^−1^ in 0.1 M K2HPO4 pH 5.0, 150 mM NaCl) was verified performing dynamic light scattering (DLS) measurements at 20 °C. 15 µl of purified GqqA suspension were measured in Helma cuvettes applying the SpectroSize 300 DLS system (Xtal Concepts, Germany).

### Small angle X-ray scattering

Small angle X-ray scattering data for GqqA were collected at 20 °C at beamline P12 (PETRA III, EMBL, Hamburg, Germany). Three different protein concentrations (4, 2 and 0.8 mg ml^−1^) were measured applying an automated robotic sample changer and a Dectris 2D photon-counting detector (PILATUS-6 M) with 3.1 m sample to detector distance. Scattering data for all three concentrations were collected, integrated, and averaged applying the SasTool software (EMBL, Hamburg, Germany) (Franke 2017. Guinier analysis and radius of gyration (R_G_) values were calculated using PRIMUS^41^. The pair distribution functions P(r) and forward scattering intensities I(0) were processed with GNOM^42^ and PRIMUS. Data collection statistics and results are summarized in TABLE S4.

### Crystallization

Initial screening of GqqA and SeGqqA was performed applying the sitting-drop vapor-diffusion method and 96-well plates utilizing a Honeybee crystallization robot (Genomic Solutions, USA) and using the commercial kit JCSG-plus (Molecular Dimensions, UK). Thin GqqA crystals were identified after 3 days with 0.8 M succinic acid at pH 7.0 as reservoir solution. To obtain larger and X-ray suitable crystals streak seeding was applied to seed a droplet of 2 µl protein solution (10 mg ml^−1^) and 2 µl reservoir solution using 24-well linbro plates (FIGURE S1, C and D). Seed suspensions were prepared according to the protocol of HAMPTON Research (HAMPTON Research, US). SeGqqA crystals were obtained in 10% PEG3350. Final crystallization conditions are summarized in TABLE S5.

### GqqA diffraction data collection, structure solution, and refinement

For native GqqA and SeGqqA X-ray diffraction data were collected at 100 K at beamline P11 (PETRA III, DESY, Hamburg). The diffraction data were indexed, integrated and scaled using the XDS software package^43^.

Initial structure solution was possible applying diffraction data of a single SeMet-labelled GqqA (SeGqqA) crystal collected at a wavelength of 0.98 Å. This crystal diffracted up to 1.7 Å resolution and diffraction data were used for single-wavelength anomalous phasing (SAD), performed using the Autorickshaw-Server^44^. Based on eight located Se-positions phasing was possible and initial electron densities allowed model building of SeGqqA applying the program Coot^45^. Subsequent refinement was performed utilizing the program Refmac5^46^. Interestingly this structure contained only two of the three GqqA protein domains.

Diffraction data to 2.5 Å resolution were collected for native GqqA at a wavelength of 1.03 Å. The obtained SeGqqA structure was used for molecular replacement to obtain initial phases of the native GqqA structure using the program Phaser^47^. The native structure was subsequently completed by building amino-acid residues 85–170 of the missing domain into the 2Fo-Fc electron density maps. The following structure refinement was performed using the program Phenix^48^. In later stages of the refinement the Phenylalanine co-factor and solvent molecules were introduced and refined. Data collection statistics and refinement results are summarized in Table 2. The refined GqqA and SeGqqA structures were deposited in the Protein Data Bank with pdb codes 7AM0 and 7ALZ.

**Table 2:** X-ray diffraction data collection, processing and refinement statistics.

	SeGqqA	GqqA
Diffraction source	P11 at PETRA III (DESY), Hamburg	P11 at PETRA III (DESY), Hamburg
Wavelength (Å)	0.9801	1.0332
Detector	Pilatus	Pilatus
Crystal-detector distance (mm)	270	479
Rotation range per image (°)	0.05	0.1
Total rotation range (°)	360	360
Exposure time per image (s)	0.1	0.1
Space group	*P*2_1_2_1_2_1_	*P*2_1_
Unit cell parameters (Å, °)	a = 60.44, b = 63.73, c = 97.34,	a = 76.87, b = 65.76, c = 102.66, β = 104.27
Resolution range (Å)	50.0–1.67 (1.78–1.67)	49.3–2.5 (2.6–2.5)
Total no. of reflections	545,065 (85,204)	234,394 (23,288)
No. of unique reflections	79,955 (12,767)	34,461 (3364)
Completeness (%)	99.7 (98.0)	99.44 (98.53)
Multiplicity	6.82 (6.67)	6.8 (6.9)
*I*/σ(*I*)	21.9 (3.36)	29.35 (3.90)
*R*_meas_	6.0 (60.9)	4.4 (52.4)
CC 1/2	0.99 (0.91)	1 (0.97)
Wilson *B*-factor (Å^2^)	19.2	57.4
**Refinement**		
R_cryst_ / R_free_	0.16 / 0.19	0.22 / 0.27
RMS bonds (Å) / angles (°)	0.022/ 2.04	0.006/1.07
Ramachandran favored (%)	98.9	96.5
Ramachandran allowed (%)	1.1	3.2
Ramachandran outliers (%)	none	0.3
Average *B*-factors (Å^2^)	22.0	71.1

Values for the outer resolution shell are given in parentheses.

### In silico ligand docking

Docking approaches were performed with the freely available docking software SwissDock^49,50^. The software searches possible target cavities in proteins by calculating the binding energies of small molecules. N-octanoyl-L-homoserine lactone was chosen as putative ligand due to previous experiments^1^ and provided in mol2 format. The GqqA dimer was used without solvent molecules but with bound L-Phe in the regulatory domain. The best hits are clustered and are visualized in Figure 8A.

### Construction of GqqA mutants

For the exchange of individual amino acids in GqqA, site-directed mutagenesis was used. For this purpose, the mutations were carried out using the phusion DNA polymerase, the construct pET21a::gqqA as template and the following mutation-specific primers:

GqqA_M1_for (5’ CGAACTTTCGTCCTTTTCGGAACAGCAGGAGATC 3’), GqqA_M1_rev (5’ TCCAGCGCACGCGCCAGT 3’), GqqA_M2_for (5’ CTCGAGCACCACCACCACC 3’), GqqA_M2_rev (5’ CCGGAAGGGCGATGCGGG 3’), GqqA_M3_for (5’ CATGCAAGGTCCGAATAGGCCCCGGCAGGCCTCGCCCGGCTGGA 3’), GqqA_M3_rev (5’ CATGCAAGGTCCGAATAGGCCC 3’), GqqA_M5_for (5’ CGGCATCATTGTCGAACTGGGACTGGACCCAG 3’), GqqA_M5_rev (5’ CGCACCTGCGCCATGGCG 3’). The GqqA_Mut4 construct was generated by using a synthesized gene fragment containing the substitution and deletion side. To remove the template DNA, the PCR product was digested with *Dpn*I (30 min 37 °C, 10 min heat inactivation at 75 °C). After digestion, a phosphate group was added to the amplicon polynucleotide phosphokinase using the manufacturer's protocol (Thermo Scientific, Bremen, Germany). With the added phosphate group, the linear amplicon could be ligated with the T4 ligase by adding 1 µL 10 mM ATP. The ligation was carried out at alternating between 10 °C and 30 °C for 60 min. A heat inactivation step was followed by heat shock transformation into *E. coli* DH5α. Positive clones were verified by sequencing and transformed into the overexpression strain *E. coli* BL21(DE3).

### In vivo QQ enzyme assays

For a functional AHL-degrading assay, the reporter strain *C. violaceum* CV026 was used as previously described and with minor modifications^1,52^. For this purpose, *E. coli* BL21 (DE3) strains containing the pET-21a::gqqA wt or the pET21a::gqqA M1-M5 mutant constructs were grown in a preculture. Fresh LB medium was inoculated with 1% (vol/vol) of the precultures and cultured at 37 °C until an OD600 of 0.6 was reached. Following this cells were induced with 0.1 mM IPTG and proteins were expressed for 16 h. After this fresh LB medium containing ampicillin and 10 µM oxo-C8-HSL was inoculated with the expression strains at a ratio of 2% (vol/vol) and incubated at 37 °C for 6 h. The culture supernatant was collected by centrifugation and 30 µL was used for bioassay. For this, a grown CV026 culture was mixed with 24-fold volume of LB agar and poured into plates. Sterile filter papers were placed on the plates. The CV026 plates with the supernatants of the cultures were incubated for 24 h at 28 °C. If CV026 cells turned purple, AI molecules were present; and if CV026 cells remain colourless, the AI molecules were degraded.

### β-lactamase activity assay

The β-lactamase activity of GqqA was determined via a disc diffusion antibiotic susceptibility test. Ampicillin 10 µg (AMP), Amoxicillin 10 µg (AML), Cefotaxime 30 µg (CTX 30) and Penicillin G 10 µg (P) sensitivity discs (Thermo Fischer Scientific, Waltham, MA, USA) were incubated for 30 min at 28 °C with 30 µL of 0.1 M potassium phosphate buffer pH 8 containing 40 µg of GqqA. A control without enzyme was included. The antibiotic susceptibility test was performed on LB agar plates with *S. aureus* cells. After overnight incubation at 37 °C the halo formation indicates a degradation of the antibiotic.

### Complementation assays with an auxotrophic strain of Escherichia coli

A complementation test was performed with the phenylalanine auxotrophic *E. coli* strain JW2580-1. The strain was obtained from the Coli Genetic Stock Center (http:// www.cgsc.biology.yale.edu) and carries an in-frame, single-gene knockout from the Keio Collection^51^. JW2580-1 was grown in LB medium overnight at 37 °C. Phenylalanine auxotrophy was verified to the complementation tests by growth on minimal medium M9 supplemented with and without L-phenylalanine (2 μM) (Sigma-Aldrich, Heidelberg, Germany). *gqqA* was amplified using the primers gqqA_comp_f (5’ ATGAACGGGGAACGCATCATCGC 3’) and gqqA comp_rev (5’ TCAGGGTTTGCGCCGGAG 3’) and the fragment was cloned into the pDrive vector. The resulting pDrive::gqqA vector was sequenced to verify the correctness of the sequence and was then transformed into JW2580-1 by electroporation. The transformed strain as well as the auxotrophic strain were grown in LB medium overnight at 37 °C and at 120 rpm; then, 5 ml of each culture was centrifuged, and cells were washed three times with M9 medium. 5 ml M9 medium with or without phenylalanine (2 µM) was inoculated with 50 µl of the washed cells and incubated up to 72 h at 37 °C.

### Substrate specificity analysis

Conversion rate (V_max_) ) and affinity (K_m_) for 3-oxo-hexanoyl-, 3-oxo-octanoyl-, 3-oxo-decanoyl-, and 3-oxo-dodecanoyl- homoserine lactones, all from Merck Life Science S.L.U. (Madrid, SPAIN), was calculated using a pH indicator assay in 96-well plates, at 30 °C and pH 8.0 in a Synergy HT Multi-Mode Microplate Reader in continuous mode at 550 nm over 20 min, as described^53^. For enzyme assays, conditions were as detailed previously: [protein]: 10 μg ml-1; [3-oxo acyl homoserine lactone]: 0–65 mM; reaction volume: 200 μl; buffer: 5 mM EPPS buffer pH 8.0 containing 0.5 mM Phenol Red; T: 30 °C; and pH: 8.0. The absorbance readings in all wells were measured immediately after the plate was gently shaken for 3 s. The rate of hydrolysis in each triplicate assay was recorded at 15 s intervals over 20 min. The absorbance readings for the reaction steady state (within the first minute of the assay) were used to generate a progress curve (arbitrary absorbance units vs. time) from which a line slope value was determined, and enzyme units calculated as described^53^. All values, in triplicate, were corrected for non-enzymatic transformation, with absence of activity defined as at least a two-fold background signal as described^53^.

## Supplementary Information

Supplementary Information.

## Data Availability

The coordinates and structure factors of crystal structures of GqqA and SeGqqA have been deposited in the PDB with accession codes 7AM0 and 7ALZ, respectively. All other data generated or analyzed during this study are included in this article and its supplementary Information files.
