# Supplementary material for: The Komagataeibacter europaeus GqqA is the prototype of a novel bifunctional N-Acyl-homoserine lactone acylase with prephenate dehydratase activity
Source: Sci Rep. 2021 Jun 10;11:12255. doi: 10.1038/s41598-021-91536-1 (PMC8192741; doi:10.1038/s41598-021-91536-1)
Supplement: Supplementary file 1 — Supplementary Information. [file 41598_2021_91536_MOESM1_ESM.pdf]

Supplementary file and information for:

The *Komagataeibacter europaeus* GqqA is the prototype of a novel bifunctional N-Acyl-homoserine lactone acylase with prephenate dehydratase activity

Nadine Werner[1], Katrin Petersen[2], Christel Vollstedt[2], Pablo Perez Garcia[2], Jennifer Chow[2], Manuel Ferrer[3], Laura Fernandes-Lopez[3], Sven Falke[1], Markus Perbandt[1], Winfried Hinrichs[4], Christian Betzel[1*] & Wolfgang R. Streit[2*]

[1] Institute of Biochemistry and Molecular Biology, Laboratory for Structural Biology of Infection and Inflammation, University Hamburg, c/o DESY, 22607 Hamburg, Germany
[2] Microbiology and Biotechnology, University Hamburg, 22609 Hamburg, Germany
[3] Institute of Catalysis, Consejo Superior de Investigaciones Científicas, 28049 Madrid, Spain
[4] Institute for Biochemistry, University Greifswald, 17487 Greifswald, Germany

Supplementary Tables

TABLE S1: Bacterial strains and plasmids used in this work.

Strain or plasmid	Relevant trait(s)[a]	Source or reference
**Strains**		
E. coli DH5α	F⁻ ϕ80d*lacZ*ΔM15 Δ(*argF-lacZYA*) U169 *endA1 hsdR17* (r_K^-, m_K^-) *supE44 thi-1 recA1 gyrA96 relA1*	55
E. coli BL21(DE3)	F− ompT hsdSB (rB − mB −) gal dcm (DE3)	Novagen, Darmstadt, Germany
E. coli JW2580-1	Phenylalanine auxotroph F− Δ(*araDaraB*)567, Δ*lacZ4787*(::rrnB-3), λ−, Δ*pheA762*::*kan, rph1*, Δ(*rhaD-rhaB*)*568, hsdR514*	*E. coli* Genetic stock Center, Yale, USA
C. violaceum CV026	Reporter strain for autoinducer I; mini-Tn5 in *cviI*	54
A. tumefaciens NTL4 (pCF218)(pCF372)	Reporter strain for AHL detection; *traI*::*lac*Z; Tet^r; Sp^r	56
**Plasmids**		
pDrive	Vector for PCR cloning, QIAGEN® PCR cloning kit; Amp^r; Km^r	QIAGEN, Hilden, Germany
pET-21a::*gqqA*	pET-21a containing the *gqqA* gene from *Komagataeibacter europaeus* CECT 8454	1
pET-21a::*gqqA*-M1	pET-21a::*gqqA* was modified by using site directed mutagenesis (T782C)	This work
pET-21a::*gqqA*-M2	pET-21a::*gqqA* was modified by using site directed mutagenesis (Δ835- 843)	This work
pET-21a::*gqqA*-M3	pET-21a::*gqqA* was modified by using site directed mutagenesis (C73T;G74C)	This work
pET-21a::*gqqA*-M4	pET-21a::*gqqA* was modified by using site directed mutagenesis (C559G; Δ562- 570;	This work
pET-21a::*gqqA*-M5	pET-21a::*gqqA* was modified by using site directed mutagenesis (A352G; C353T; C354C)	This work

TABLE S2: Amino acid mixture applied for expression in M9 media

10 mg ml^{-1}	L-Lysine
10 mg ml^{-1}	L-Phenylalanine
10 mg ml^{-1}	L-Threonine
5 mg ml^{-1}	L-Isoleucine
5 mg ml^{-1}	L-Leucine
5 mg ml^{-1}	L-Valine

TABLE S3: ESI-MS-MS substrate and product mass analyses

modus	molecule	mass [g/mol]	ion
positive	DMSO	101.00	$[M + Na]^+$
	HSL	134.08	$[M + MeOH + H]^+$
	DMSO	179.02	$[2M + Na]^+$
	3-oxo-C8-HSL	242.14	$[M + H]^+$
	3-oxo-C8-HSL	264.12	$[M+ Na]^+$
	3-oxo-C8-HS	296.15	$[M + MeOH + Na]^+$
	3-oxo-C8-HSL	537.28	$[2M + 2MeOH + Na]^+$
negative	3-oxo-C8-HSL	240.12	$[M - H]^-$
	3-oxo-C8-HSL	272.15	$[M +MeOH - H]^-$
	3-oxo-octanoic acid	157.09	$[M - H]^-$

TABLE S4: SAXS data collection

Beamline	EMBL Beamline P12, PETRA III, DESY, Hamburg
Wavelength (Å)	1.24403
Exposition time (s)/ Exposition period (s)	0.045/ 0.05
Detector distance (m)	3.0
Sample concentration range (mg ml^{-1})	0.9 – 4.2
Temperature (K)	293
**Structural Parameters**	
R_g (nm), calculated from Guinier approximation	2.69
D_{max} (nm), calculated from *p(r)* Function	9.4
Molecular weight (kDa), calculated from *p(r)* function	58.37

TABLE S5: Crystallization conditions

Final crystallization conditions	GqqA	SeGqqA
Method	Hanging-drop vapor diffusion	Sitting-drop vapor diffusion
Plate type	24-well linbro plate	96-well MRC2 plate
Temperature (K)	291	291
Protein concentration (mg/ml)	10	10
Buffer composition of protein solution	0.1 M K_2HPO_4 pH 5.0, 150 mM NaCl	0.1 M K_2HPO_4 pH 7.0, 150 mM NaCl
Composition of reservoir solution	0.67 M succinic acid pH 6.0	10 % PEG3350
Volume and mixing ratio of droplets (µl)	4 (2+2)	0.6 (0.3+0.3)
Reservoir volume (µl)	500	50

Supplementary Figures

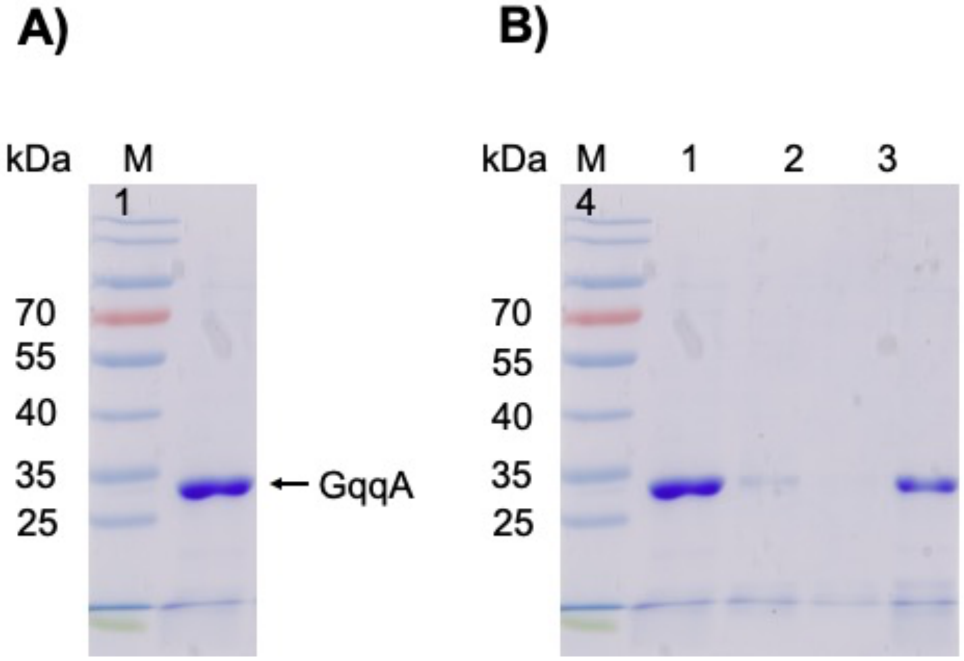

FIGURE S1: Biochemical characterization of GqqA (A) Recombinant and purified GqqA; 12% SDS-PAGE of pure GqqA protein (30.5 kDa). Lane M contains molecular weight markers, labelled with molecular weight in kDa (Unstained Protein Molecular Weight Marker, Thermo Scientific); **(B)** Non cropped version of A.

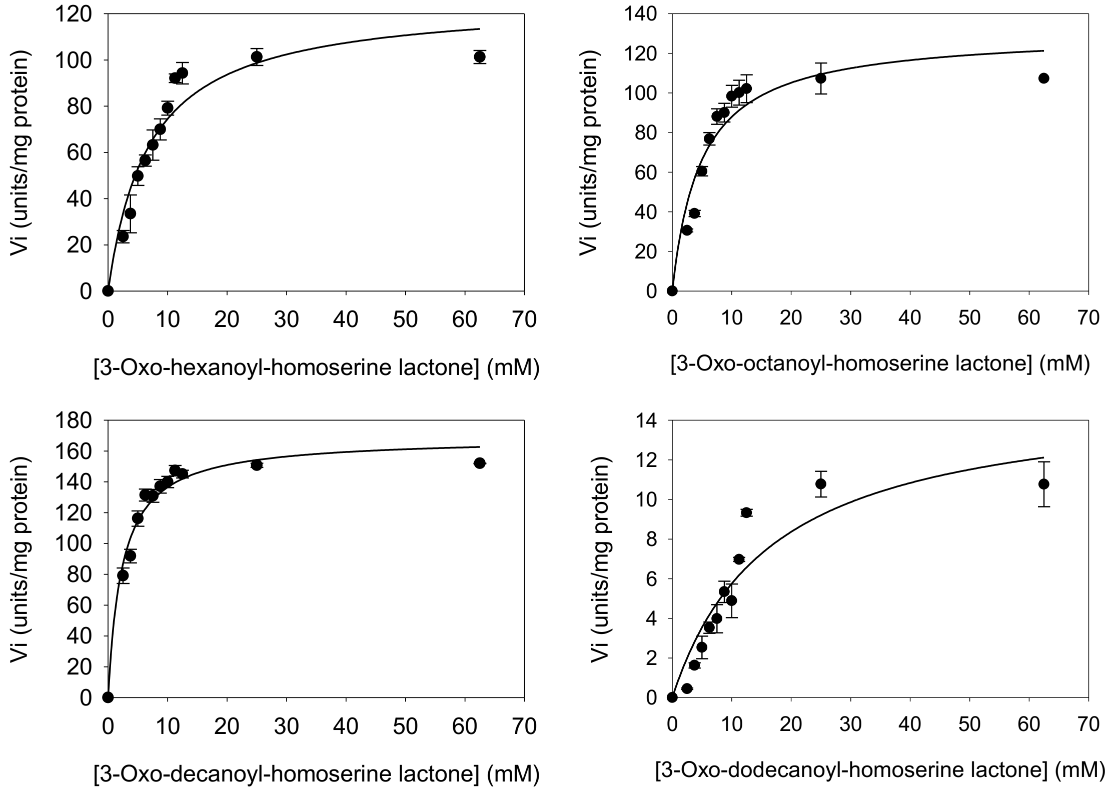

FIGURE S2: The Michaelis-Menten fits for K_m and V_{max} determinations for the conversion of 3-oxo-acyl homoserine lactones by GqqA. Graphics were created with SigmaPlot version 14.0, with data being fitted to Dynamic Fit Wizard model (one site saturation).

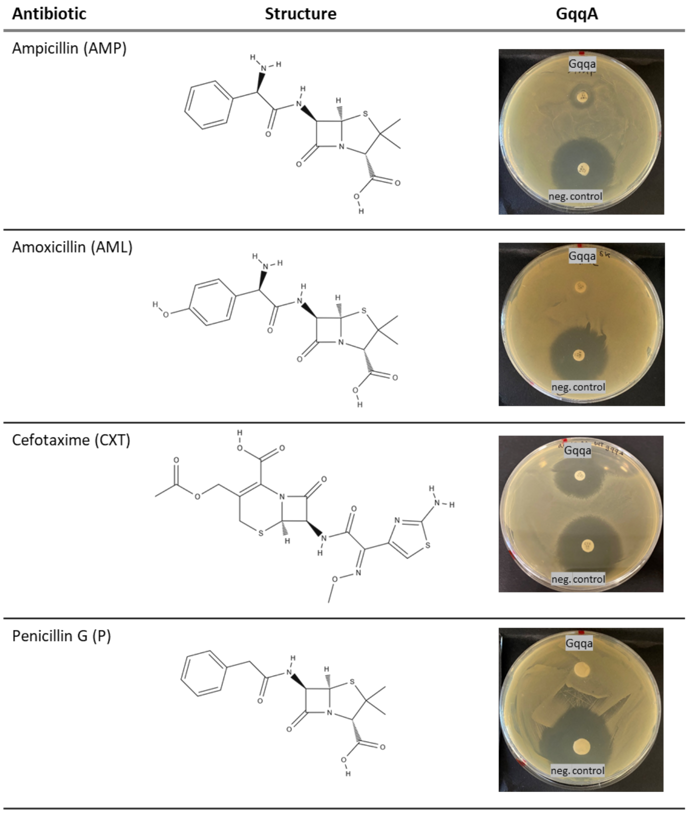

FIGURE S3: Acylase activity of GqqA. The β-lactamase activity of GqqA was determined via a disc diffusion antibiotic susceptibility test. Ampicillin 10 µg (AMP), Amoxicillin 10 µg, (AML) Cefotaxime 30 µg (CTX 30) and Penicillin G 10 µg (P) sensitivity discs (Thermo Fischer Scientific, Waltham, MA, USA) were incubated for 30 min at 28°C with 30 µL of 0.1 M potassium phosphate buffer pH 8 containing 40 µg of GqqA. A control without enzyme was included. The antibiotic susceptibility test was performed on LB agar plates with *S. aureus* cells. The degradation of the antibiotic is visible in a lack of halo formation.

FIGURE S3

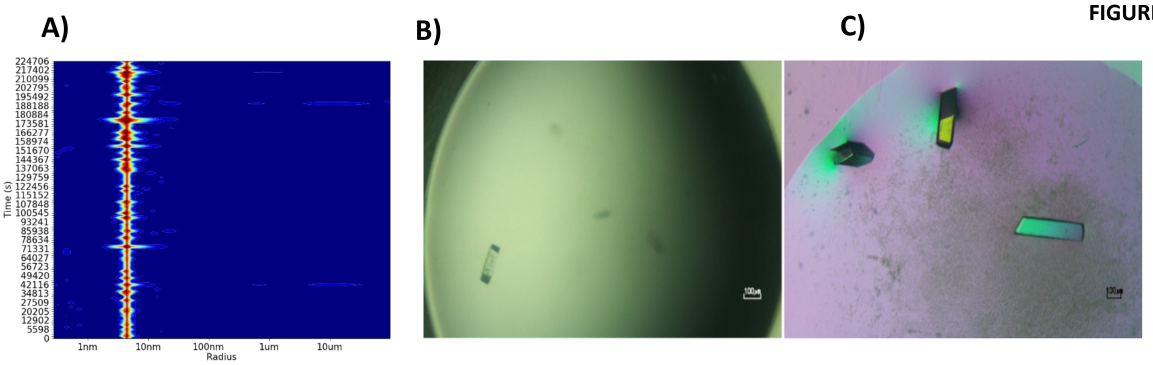

FIGURE S4: **(A)** Long-term DLS measurements; DLS measurement of GqqA (3.6 mg ml^{-1}) revealed a hydrodynamic radius of 3.9 ± 0.1 nm, corresponding to a GqqA dimer (61 kDa). The relative abundance of particles is color-coded from dark blue (low) to dark red (high); **(B)** Crystals of SeGqqA and **(C)** GqqA crystals obtained after seeding experiments).
